# Inter-professional in-situ simulated team and resuscitation training for patient safety: Description and impact of a programmatic approach

**DOI:** 10.1186/s12909-015-0472-5

**Published:** 2015-10-29

**Authors:** Katja Zimmermann, Iris Bachmann Holzinger, Lorena Ganassi, Peter Esslinger, Sina Pilgrim, Meredith Allen, Margarita Burmester, Martin Stocker

**Affiliations:** Department of Paediatrics, Children’s Hospital Lucerne, CH-6000 Lucerne 16, Switzerland; University Children’s Hospital Berne, Inselspital, CH-3000 Bern, Switzerland; The Royal Children’s Hospital, Flemington Road, Parkville, VIC 3052 Australia; Royal Brompton Hospital, Sydney Street, London, SW3 6NP UK

**Keywords:** In-situ simulation, Team training, Resuscitation, Programmatic approach, Implementation strategy, Kern’s six steps

## Abstract

**Background:**

Inter-professional teamwork is key for patient safety and team training is an effective strategy to improve patient outcome. In-situ simulation is a relatively new strategy with emerging efficacy, but best practices for the design, delivery and implementation have yet to be evaluated. Our aim is to describe and evaluate the implementation of an inter-professional in-situ simulated team and resuscitation training in a teaching hospital with a programmatic approach.

**Methods:**

We designed and implemented a team and resuscitation training program according to Kern’s six steps approach for curriculum development. General and specific needs assessments were conducted as independent cross-sectional surveys. Teamwork, technical skills and detection of latent safety threats were defined as specific objectives. Inter-professional in-situ simulation was used as educational strategy. The training was embedded within the workdays of participants and implemented in our highest acuity wards (emergency department, intensive care unit, intermediate care unit). Self-perceived impact and self-efficacy were sampled with an anonymous evaluation questionnaire after every simulated training session. Assessment of team performance was done with the team-based self-assessment tool TeamMonitor applying Van der Vleuten’s conceptual framework of longitudinal evaluation after experienced real events. Latent safety threats were reported during training sessions and after experienced real events.

**Results:**

The general and specific needs assessments clearly identified the problems, revealed specific training needs and assisted with stakeholder engagement. Ninety-five interdisciplinary staff members of the Children’s Hospital participated in 20 in-situ simulated training sessions within 2 years. Participant feedback showed a high effect and acceptance of training with reference to self-perceived impact and self-efficacy. Thirty-five team members experiencing 8 real critical events assessed team performance with TeamMonitor. Team performance assessment with TeamMonitor was feasible and identified specific areas to target future team training sessions. Training sessions as well as experienced real events revealed important latent safety threats that directed system changes.

**Conclusions:**

The programmatic approach of Kern's six steps for curriculum development helped to overcome barriers of design, implementation and assessment of an in-situ team and resuscitation training program. This approach may help improve effectiveness and impact of an in-situ simulated training program.

## Background

Improving patient safety is imperative for every health care organization [1]. Inter-professional teamwork is key for patient safety and team training is an effective strategy to improve patient outcome [2–4]. Nurses and doctors working in acute care experience critical clinical events of rapidly deteriorating patients in need of cardiopulmonary support. Mismanagement of deteriorating patients is the most common failure reported in patient-safety-related Hospital deaths in England [5]. Recent reports suggest that hospital staff members feel inadequately prepared, perceive deficits and a high level of anxiety when managing cardiopulmonary arrest situations [6–8]. Optimal management of these events requires knowledge, technical skills, teamwork and can be enhanced by inter-professional training of hospital staff. Recently published studies show a good impact of simulation training for technical as well as non-technical skills [9–17]. In addition to teamwork and individual skills, patient safety can be improved through the identification and correction of latent safety threats [18–20].

In-situ simulation is a relatively new strategy with emerging data to support its efficacy. Recently published reports have discussed how to implement efficient simulated team training, [15, 21] but best practices for the design, delivery and implementation of an in-situ program have yet to be established [21–26]. A programmatic approach to training and assessment based on system thinking is required for a sustained improvement of team performance and patient safety [21, 27]. We developed an inter-professional in-situ simulated team and resuscitation training program (iSTaRT) for patient safety according to the framework for curriculum development by Kern [28]. The purpose of this prospective study is to describe the development, implementation and impact of an inter-professional in-situ simulated team and resuscitation training program designed with a programmatic approach based on Kern's six steps.

## Methods

### Setting

The Children's Hospital of Lucerne is a tertiary teaching hospital in the heart of Switzerland with general paediatric and paediatric surgery departments. Before implementation of iSTaRT, regular continuing education for doctors and nurses included lectures, journal club, small learning groups and low-fidelity simulation (paediatric and neonatal resuscitation). Driven by a small highly motivated group of staff members, an inter-professional project group was launched to design and implement a simulation-based team and resuscitation training program to improve patient safety during future critical events of rapid deteriorating patients in need of cardiopulmonary support at the Children’s Hospital of Lucerne. The group was led by 3 staff members with experience in simulation training (KZ, IBH, MS), whereas other group members did not have knowledge regarding simulation-based training. Two co-authors of the study with expert knowledge in designing and implementation of an in-situ simulated team training program were invited to be external consultants (MA, MB) [22]. The aim was to select a programmatic approach for curriculum development to improve the chance for successful design, implementation and acceptance of the training program and to prospectively evaluate the impact [21, 27]. According to published evidence there is a 6- to 12-month learning curve in the implementation of a simulated training program and therefore we selected a 2-year study period [22].

### Programmatic approach for curriculum development

**Fig. 1:**
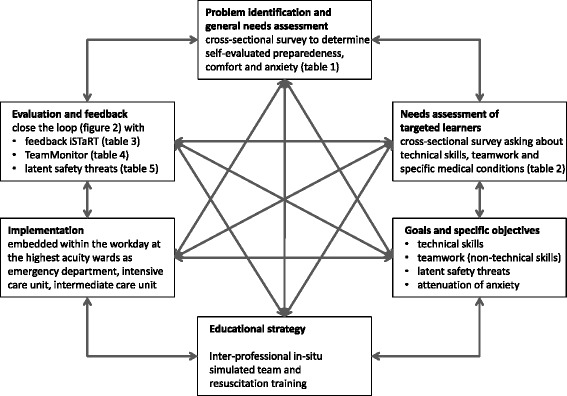
Programmatic approach. Programmatic approach using the six-step approach of Kern for curriculum development [28]

Kern’s framework for curriculum development for medical education was selected due to the short, practical and general approach [28]. Grounded in systems thinking it assumes curriculum development is a continuous, dynamic and interactive process that may start at different steps, is not always linear and the process never really ends [28]. Systems thinking states that changes in a part of a system will always cause the whole system to change, and that change developed through a programmatic approach is more likely to be sustainable [29, 30]. The six-step approach of Kern includes: i) general needs assessment to identify the problem, ii) focused needs assessment of targeted learners, iii) goals and specific measurable objectives, iv) educational strategies, v) implementation, vi) evaluation and feedback to stakeholders (Fig. 1).

**Fig. 2:**
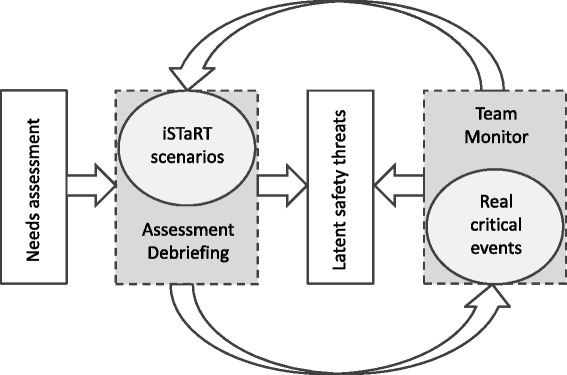
Close the loop between training and reality. Close the loop between simulated training sessions, experienced real critical events and system-based factors (latent safety threats)

Kern’s step 1 and 2: General and specific needs assessments were conducted as independent cross-sectional surveys. The purpose of the general needs assessment was to determine self-evaluated preparedness, comfort and anxiety regarding performance in a possible future critical event among inter-professional staff members at the Children's Hospital Lucerne (5-item questionnaire). Classification of answers was done with a 5-point Likert scale. The specific needs assessment was a questionnaire including pre-defined topics and an option for free-text responses asking regarding training needs of technical skills, non-technical skills (teamwork) and specific medical conditions.

Kern’s step 3: According to Kern's six-step approach for curriculum development we used the results of the needs assessments to define the goals and objectives of the training program [28]. Technical skills as correct bag and mask ventilation, cardiac compressions and knowledge of emergency medication; non-technical skills as role allocation, leadership and communication (close-loop communication) and attenuation of anxiety regarding participation in future critical events were defined as learning objectives. In addition, system-based factors are well known as latent safety threats in every health care organization and there is evidence that system redesign may help to reduce errors in hospitals [31]. Therefore, detection of latent safety threats - defined as factors that make errors more likely or more dangerous [32] - was selected as a specific measurable outcome of our training program. In order to assess the impact of the program on the detection of latent safety threats all debriefing sessions included the question “Do you feel that specific system-based factors had an impact on your (or the teams) performance?” Feedback forms after the training session and after real events also prompted participants to consider latent safety threats and document possible solutions. Latent threats identified were shared with department heads. All implemented changes made due to identification of system-based factors during an iSTaRT session were prospectively recorded.

Kern’s step 4: We developed iSTaRT based on the concept of in-situ simulation-based crisis resource management (CRM) training as educational strategy [21–25]. We used a number of established learning theories to optimize our conceptual framework [24]. “Challenge participants to the edge”, “facilitate critical reflection” and “motivate with reality and context” were guiding statements for the facilitators. Each training session consisted of an introduction to CRM principles, a simulated scenario and a structured debrief over 2 h. Simulated scenarios were derived from real critical events and according to the collected needs assessment data. To provide care as realistically as possible participants were asked to perform as in reality including airway management, cardiopulmonary resuscitation, defibrillation, drawing-up and administration of medications. Initially, we used a low-fidelity mannequin (Neonatal Resuscitation Baby, Laerdal^®^) and, after 6 sessions a high-fidelity mannequin (Pediatric HAL 1 year, Gaumard^®^) aiming to further improve the realistic context of the training. The replacement was mainly in response to the good experience of some of the facilitators with high fidelity simulation in past [22]. Purchase of the high-fidelity mannequin was possible thanks to managerial buy-in with the project. The structured debriefing lasted around 1 h and was mainly based on the 3D model of debriefing and debriefing with good judgment [23, 24, 33, 34]. Facilitators of the debriefing guided and empowered participants to reflect openly on their own behaviors, to challenge own beliefs and frames and to conceptualize new principles and actions. Facilitators of the debriefing were trained within the PAEDSIM train-the-trainer programme (www.paedsim.org).

Kern’s step 5: Monthly training sessions were embedded within the work day of participants. Usually, the session was realized before or at the end of participant’s shift facilitating participation and raising acceptance. Due to restricted resources, we commenced the iSTaRT program just in our highest acuity wards with the highest risk of occurrence of critical clinical events: the emergency department, combined neonatal and paediatric intensive care unit, intermediate care unit. According to the principles of in-situ simulation all training sessions were completed on the regular unit with real inter-professional team members

Kern’s step 6: Assessment of training sessions (Fig. 2): After scenario and structured debriefing, all participants received an anonymous feedback form to assess the training session and its impact on their clinical practice. The feedback form contained six questions answered on a 5-point Likert scale [22]. Van der Vleuten’s framework of a programmatic approach to evaluate multiple sets of assessment longitudinally guided our assessment strategy of real events during the second half of the reported time of the programme [35]. TeamMonitor, a modified version of the Mayo High Performance Teamwork Scale published with a good reliability (kappa = 0.86) and content validity for CRM training, was used as the assessment tool [36]. TeamMonitor was selected due to the reported feasibility and low use of resource requirements compared to video analysis or expert observation [36]. As a 9-item team-based self-assessment tool capturing aspects of leadership, role clarity, communication, resource utilization and situational awareness, every item is scaled as 0 (never/rarely), 1 (inconsistently), 2 (consistently) or not applicable. The definition of training needs was selected after discussion within the facilitator group. We determined that when a desired behavior was experienced as rare or inconsistent by one third or more of participants, then further improvement was required. Therefore, items scored ≤1 in more than 33 % of response were considered to be targets for training, and in more than 50 % to be require urgent attention in further training sessions.

A combination of quantitative and qualitative methodology was utilised. Descriptive statistics were used for overall responses. General needs assessments and feedback forms (5-point Likert scales) were consistent with independent ordered categories. Scores ≤2 were defined as very low (score 1) or low (score 2), scores ≥4 as high (score 4) or very high (score 5) competence. Scores ≥4 were designated as desirable and/or good impact, scores ≤2 were problematic and/or showing no impact. Physicians and nurses responses were compared using chi-square tests with two degrees of freedom. Items scoring ≤1 in the TeamMonitor assessment tool were compared for single as well as for all events together using chi-square test with two degree of freedom. Pearson’s p value <0.05 was considered as statistically significant with a confidence interval at 95 %. All authors had full access to all data in the study and take responsibility for the integrity and accuracy of the analysis. Anonymous questionnaires, as a standard part of an educational program, did not require ethical approval according to the institute’s ethical committee, as well as according to the ethical guidelines of the British Educational Research Association (BERA) [37].

## Results

### Needs assessment

In August 2011, 121 staff members (48 % of all staff members) participated in the general needs assessment. 82 (41 % of nursing staff) of the 121 respondents were staff nurses and 39 (71 % of physicians) were paediatricians including subspecialists and paediatric surgeons. Results of the general needs assessment are shown in Table 1. Whilst the majority of both professions evaluated their competence and preparedness to detect a deteriorating patient highly, less than 50 % of surveyed staff felt confident to manage a critical deteriorating patient in need of cardiopulmonary support with slightly more nurses feeling confident compared to medical staff.

**Table 1:** Results general needs assessment. Self-evaluated preparedness, comfort and anxiety regarding performance in a possible future critical event among inter-professional staff members at the Children’s Hospital Lucerne

	Total (*n* = 121)		Nurses (*n* = 82)		Physicians (*n* = 39)		Chi^2^ *p*-value	
	Score ≤2	Score ≥4	Score ≤2	Score ≥4	Score ≤2	Score ≥4		
Recognition of deterioration^a^	3 (2 %)	94 (78 %)	3 (4 %)	65 (79 %)	0	29 (74 %)	ns	ns
Management of critical events^a^	37 (31 %)	23 (19 %)	21 (26 %)	19 (23 %)	16 (41 %)	4 (10 %)	ns	ns
Competence of current team^a^	26 (21 %)	41 (34 %)	10 (12 %)	31 (38 %)	16 (41 %)	10 (26 %)	<.01	ns
Role allocation^a^	29 (24 %)	35 (29 %)	15 (18 %)	25 (30 %)	14 (36 %)	10 (26 %)	<.05	ns
Anxiety^b^	42 (35 %)	18 (15 %)	31 (38 %)	14 (17 %)	11 (28 %)	4 (10 %)	ns	ns

Chi^2^ between nurses and physicians

Classification of answers was done with a 5-point Likert scale

^a^Score ≤2: very low or low competence; Score ≥4: high or very high competence; ^b^Score ≤2: very high of high anxiety; Score ≥4: low or no anxiety

ns = not significant

In October 2011, 124 staff members participated in the specific needs assessment (49 % of all staff members). 32 were physicians (58 % of all staff physicians: paediatricians including subspecialists and paediatric surgeons of all level of expertise), 92 nurses (46 % of nursing staff). The result of the specific needs assessment regarding knowledge and technical skills, specific clinical situations and management of critical events are shown in Table 2. In contrast to the results of the general needs assessment, nurses perceived higher needs for education, reaching statistical significance for cardiac arrest/defibrillation, emergency medications/algorithms, multiple trauma and team concept/organisation/crisis resource management (CRM) principles (Table 2).

**Table 2:** Results specific needs assessment. Questionnaire including pre-defined topics and an option for free-text responses asking regarding training needs of technical skills, non-technical skills (teamwork) and specific medical conditions among inter-professional staff members at the Children’s Hospital Lucerne. Only topics reported by more than 50 % of participants (nurses and physicians) are shown

Knowledge and technical skills	Total (*n* = 124)	Nurses (*n* = 92)	Physicians (*n* = 32)	Chi^2^
Airway management	81 (65 %)	63 (68 %)	18 (56 %)	ns
ᅟBag and mask ventilation	69 (56 %)	55 (60 %)	14 (44 %)	ns
ᅟCardiac massage	67 (54 %)	54 (59 %)	13 (41 %)	ns
ᅟDefibrillation	70 (56 %)	57 (62 %)	13 (41 %)	*p* < .05
ᅟEmergency medications	101 (81 %)	82 (89 %)	19 (59 %)	*p* < .01
Specific clinical situations				
ᅟRespiratory problems	72 (58 %)	54 (59 %)	18 (56 %)	ns
ᅟRespiratory arrest	81 (65 %)	61 (66 %)	20 (62 %)	ns
ᅟCardiac arrest	76 (61 %)	62 (67 %)	14 (44 %)	*p* < .05
ᅟShock	77 (62 %)	59 (64 %)	18 (56 %)	ns
ᅟMultiple trauma	66 (53 %)	58 (63 %)	8 (25 %)	*p* < .01
Management of critical events				
ᅟTrauma room management	69 (56 %)	52 (57 %)	17 (53 %)	ns
ᅟPALS Algorithms	77 (62 %)	64 (70 %)	13 (41 %)	*p* < .01
ᅟTeam concept	84 (68 %)	69 (75 %)	15 (47 %)	*p* < .01
ᅟCRM	73 (59 %)	59 (64 %)	14 (44 %)	*p* < .05
ᅟOrganization	88 (71 %)	70 (76 %)	18 (56 %)	*p* < .05
ᅟCommunication	82 (66 %)	64 (70 %)	18 (56 %)	ns
ᅟError prevention	83 (67 %)	64 (70 %)	19 (59 %)	ns

Chi^2^ between nurses and physicians

ns = not significant

CRM = Crisis resource management, PALS = paediatric advanced life support

### Feedback responses

95 staff members participated in 20 simulation scenarios which took place on the 3 units between January 2012 and February 2014. 50 of the 95 participants (53 %) were staff nurses and 45/95 (47 %) were physicians (paediatricians including subspecialists, paediatric surgeons and anaesthetists). Two to three physicians and two to four nurses participated in each scenario. 86/95 (91 %) participants returned their questionnaires. Results of the feedback questionnaire regarding self-perceived impact on improvement on teamwork, technical skills, knowledge and anxiety for future critical events are shown in Table 3. There was no statistical significant difference between nurses and physicians.

**Table 3:** Participants self-perceived impact of an iSTaRT-session

	Total (*n* = 86)		Nurses (*n* = 41)		Physicians (*n* = 45)		Chi^2^ *p*-value	
	Score ≤2	Score ≥4	Score ≤2	Score ≥4	Score ≤2	Score ≥4		
Improvement teamwork^a^	0	85 (99 %)	0	40 (97 %)	0	45 (100 %)	ns	ns
Improvement technical skills^a^	1 (1 %)	69 (80 %)	0	36 (88 %)	1 (2 %)	33 (74 %)	ns	ns
Improvement knowledge^a^	1 (1 %)	77 (90 %)	0	37 (90 %)	1 (2 %)	40 (89 %)	ns	ns
Improvement anxiety^a^	1 (1 %)	68 (79 %)	1 (2 %)	35 (85 %)	0	33 (74 %)	ns	ns

Chi^2^ between nurses and physicians

^a^Score ≤2: no or low impact; Score ≥4: high or very high impact

ns = not significant

Between January 2013 and February 2014, 8 real critical events occurred in the emergency department. 35 involved team members (nurses and physicians including paediatricians, paediatric surgeons and paediatric intensivists) answered the TeamMonitor questionnaire regarding team performance (mean 4.4 members/event; minimal 2, maximal 6 members). Understanding of his/her role and communication (loud verbalizing of activities involving the patient and closed loop communication) were assessed as urgent gaps with >50 % scores ≤1 (Table 4). Leadership (recognition of the leader and balance between command authority and team member participation) and response to potential errors or complications were assessed as gaps with >33 % scores ≤1, whereas the other 3 areas were perceived as having been performed well. The difference between items assessed as urgent gaps compared to items with good performance was statistically significant.

**Table 4:** Teamwork assessment in real critical events (TeamMonitor) [36]

Items		Not applicable	Number of score ≤ 1	Need for training
1	Do you feel that leader was recognized by all team members ?	1/35	14/34	yes
		(3 %)	(41 %)	
2	Do you think the leader assured maintenance of an appropriate balance between command authority and team member participation ?	3/35	11/32	yes
		(9 %)	(34 %)	
3	Do you feel that each team member demonstrated clear understanding of his/her role ?	1/35	20/34	urgent
		(3 %)	(59 %)*	
4	Do you think the team prompted each other to attend to all significant clinical indicators throughout the scenario ?	0/35	10/35	none
		(−)	(29 %)*	
5	Do you think team members verbalized their activities loud when they were actively involved with the patient ?	0/35	31/35	urgent
		(−)	(89 %)*	
6	Do you feel that the team members repeated back or paraphrased instructions and clarifications to indicate that they heard them correctly ?	0/35	31/35	urgent
		(−)	(89)*	
7	Do you feel that disagreement of conflicts among team members were adressed without a loss of situation awareness ?	16/35	3/19	none
		(46 %)	(16 %)*	
8	Do you think roles were shifted to adress urgent or emergent events when appropriate ?	15/35	5/20	none
		(43 %)	(25 %)*	
9	Do you think team members responded to potential errors or complications with procedures that avoided the error or complication ?	15/35	8/20	yes
		(43 %)	(40 %)	

TeamMonitor (team-based self-assessment tool for teamwork: modified Mayo High Performance Teamwork Scale): 0 = never/rarely; 1 = inconsistently; 2 = consistently

*Difference of urgent-gap items 3, 5 and 6 to no-gap items 4, 7 and 8 are statistically significant (*p* < 0.05)

### Latent safety threats and system changes

A total of 23 different latent safety threats were detected during both the iSTaRT sessions (1.1 per session) and following real events (1.4 per event). Identification of these latent threats resulted in implementation of new guidelines, workshops and equipment changes (selected examples in Table 5). Changes were possible within a short period of time due high motivation of study participants and support from heads of department/senior management team.

**Table 5:** Latent safety threats. Selected sample of identified latent safety threats and implemented changes

Latent threat identified	Implemented change
Insufficient knowledge and skills to use an intraosseous needle	Regular workshops for intraosseous needle placement
Deficient team performance regarding leadership, role allocation and resuscitation calls	Revision of emergency guidelines for critical events focusing on these aspects
Insufficient handover (lack of information, information unclear, not structured)	New structured handover guidelines, checklist to improve adherence, audit process supervising implementation
Fixation resulting in a loss of awareness of time during critical events at the emergency department	New timers at the emergency department with a high visibility
Failures and time delay to drawn up adrenaline (epinephrine)	Implementation of pre-drawn up, ready to use adrenaline syringes fabricated by the pharmacology department
Insufficient resuscitation equipment at the wards and the emergency department for adults	Implementation of a resuscitation bag for children with the most important equipment

## Discussion

We designed our in-situ simulated inter-professional team training using a programmatic approach according to the six steps of Kern's curriculum development. This approach helped to overcome important barriers to curriculum design. Our general needs assessment revealed the need for change and played a key role in getting senior management and stakeholder engagement. The specific needs analysis helped to align objectives to the learners needs and may have played a role achieving a good impact. Using in-situ simulation as an educational strategy revealed latent safety threats and helped to change system-based risk factors improving patient safety. The identification and correction of latent threats played a key role in maintaining senior management support for the program. TeamMonitor as a team-based self-assessment tool was successfully applied in real critical events, reduced the gap between training session and reality and identified specific areas to further target training.

A recently published review provides insight regarding how in-situ simulation is currently being used, implemented, and evaluated with the following key findings: i) Formal needs analysis methods are rarely used, ii) in-situ simulation trainers are rarely trained for assessment and feedback, iii) programmes typically address multiple levels of performance, iv) performance measurement practices are rarely formal or rigorous and v) evaluation practices are currently poor [21]. The conclusions are that evidence regarding in-situ simulation efficacy is still emerging and that practical programme planning strategies are needed [21].

We conducted a formal general needs assessment. Our general needs assessment showed that a substantial number of staff members at the Children's Hospital Lucerne felt inadequately trained regarding management of a future critical clinical event and reported to have a high level of anxiety. Lack of adequate training can be responsible for feelings of being unprepared, overwhelmed, and a heightened anxiety about committing errors during the critical clinical event. These findings are consistent with the literature and a strong argument for revision of existing training strategy [6–8]. This information played a key role in getting senior management and stakeholder engagement. Emerging evidence indicates that leadership support at all levels is mandatory for implementation and sustained success of new training methods [38–41]. Engagement of the leadership team also facilitated the purchase of a high-fidelity mannequin to improve context and environment of the training session. A high realism simulated environment may increase functional fidelity creating a powerful learning experience [42]. The specific needs analysis defined the program goals and objectives by allowing the targeted learners to order the set the possible subject matter to include in the curriculum [28]. Participants scoring of perceived benefits of the program may be high because the curriculum was well aligned to learner needs. The results of participants’ self-assessed confidence and self-efficacy is in accordance with other recent publications using in-situ simulated team training [22, 26, 43].

Using in-situ simulation, different levels of learning can be addressed: individual, team-based, unit-based and organizational learning [15, 21]. The possibility to address organizational learning and system-based issues is one of the key benefits of in-situ simulation compared to simulation-center based learning. Observations during and reflected topics by participants and facilitators within the facilitated debriefing after simulated events revealed latent safety threats and had an impact on many system-based factors. This is in line with the literature using in-situ simulation as a tool to reveal latent safety threats or to design new clinical procedures or environments [44–47]. Interestingly, even more latent safety threats (1.4 per event) were detected through facilitated reflection by self assessment after experienced real critical events. The identification and correction of latent threats played a key role in maintaining senior management ongoing support for the program. Recording of latent safety threats and implementation of changes of system-based factors are at the second highest level of measurement effectiveness adapted from Kirkpatrick’s levels of effectiveness [48]. Applying Kern’s six steps approach for curriculum design may have helped to identify important objectives for improvement and facilitated sustained change through multiple impacts at different levels as personal, process and data generation. There are some paediatric studies reporting an impact on effective health care outcomes after simulated team training. Andreatta reported that a simulation-based mock code program significantly improved paediatric cardiopulmonary arrest survival over a period of 3 consecutive years [49]. Theilen reported an improved recognition and management of deteriorating patients coincided with significantly reduced hospitality mortality with implementation of an in-situ simulated team training comparable to our program [50]. Similar, implementation of an interdisciplinary, post-event debriefing program was associated with improved survival with favourable neurologic outcome in a study at the Children’s Hospital of Philadelphia [51].

The evaluation of team performance during real critical events with team-based self-monitoring using TeamMonitor gave additional information regarding behaviours of inter-professional teams in reality [36]. This approach helps to close the gap between training session and reality. Video analysis and observation by third party are alternative approaches with the benefit of more objective data, but resource intensive and difficult ethically [52]. The analysis of our results of TeamMonitor shows that the use of TeamMonitor after real critical events is feasible. Interestingly, despite the small number of scores, there was a significant difference between items of good and low performance. Communication, role allocation and leadership were perceived as on-going training needs. Consistent with a programmatic design, this information further directed our simulated team training iSTaRT curriculum. Importantly, communication and leadership are essential for efficient and successful teamwork [53, 54].

There are several limitations of our study: First, due to the single centre, observational study setting it is not possible to inform regarding cause and effect of our programmatic approach. Second, due to the short study period, the low occurrence of critical events with mortality and the limited introduction of the program to only our highest acuity wards we were not able to assess the impact of the program in regard to patient outcome. Third, we were not able to analyze the direct impact of iSTaRT on the performance in real critical events due to the anonymous team-based assessment.

## Conclusions

A programmatic approach of curriculum development may help to improve the implementation, effectiveness and impact of a new educational program. The application of Kern’s framework for curriculum development was feasible, helped achieve stakeholder buy-in and guided the successful implementation of the program to achieve a good impact. TeamMonitor as team-based self-assessment tool was successfully applied in real critical events and identified specific areas to further target training. Detection of latent safety threats during in-situ simulated training sessions and during real critical events helped to change system-based risk factors improving patient safety.
